# Fabella Fractures after Total Knee Arthroplasty with Correction of Valgus Malalignment

**DOI:** 10.1155/2016/4749871

**Published:** 2016-06-01

**Authors:** Thomas Christian Kwee, Ben Heggelman, Robert Gaasbeek, Maarten Nix

**Affiliations:** ^1^Department of Radiology and Nuclear Medicine, University Medical Center Utrecht, Heidelberglaan 100, 3584 CX Utrecht, Netherlands; ^2^Department of Radiology, Meander Medical Center, Amersfoort, Netherlands; ^3^Department of Orthopedics and Trauma, Meander Medical Center, Amersfoort, Netherlands

## Abstract

The incidence of fabella fractures is considered to be extremely low. This report presents two patients with femorotibial osteoarthritis and considerable preoperative valgus malalignment, who developed a fracture of the fabella (as demonstrated by radiography) after total knee arthroplasty with intraoperative correction of the valgus malalignment. Special attention should be paid to the fabella for not missing a fabella fracture in these patients.

## 1. Introduction

**Figure 1:**
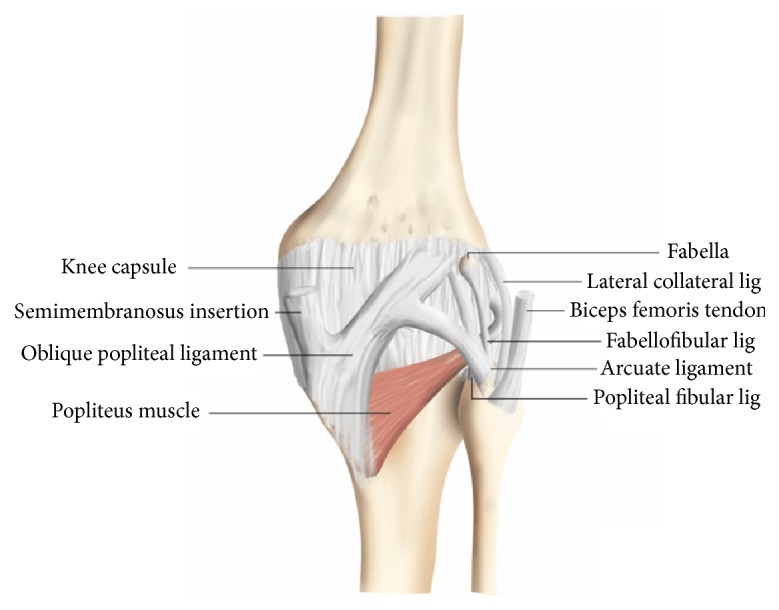
Posterolateral ligamentous complex of the knee including arcuate ligament, fabellofibular ligament, popliteal-fibular ligament, lateral collateral ligament, and biceps femoris tendon (case courtesy of Dr. Frank Gaillard, http://radiopaedia.org/). The fabella is located in the posterior aspect of the knee where lines of tensile stress intersect [14]. It articulates with the posterior part of the articular surface of the lateral femoral condyle and is embedded in the muscular fibres of the gastrocnemius muscle [14]. Anteriorly the fabella is bordered by the posterior capsule of the knee joint and posteriorly it is situated at the endpoint of the oblique popliteal ligament and the lateral gastrocnemius tendon [14]. In addition, the fabellofibular ligament runs to its distal insertion at the styloid process of the fibular head [14].

The fabella (Latin for “little bean”) is a sesamoid bone that is visible at radiography in approximately 10–30% of the general population and is found bilaterally in most cases [1]. However, according to human cadaver studies, the frequency of the fabella is higher, up to 66% [2], due to the fact that purely fibrocartilaginous fabellae are invisible at radiography. Mechanical stresses and intrinsic genetic factors are thought to play a role in the development of the fabella [1, 3]. The vast majority of fabellae are located in the tendon of the lateral head of the gastrocnemius muscle and often directly articulate with the posterior surface of the lateral femoral condyle [1]. This sesamoid bone, when present, also has a central position in the posterolateral ligamentous complex (Figure 1) [2, 4]. At present, the main clinical relevance of the fabella is to recognize its presence and to not confuse it with a loose body. However, the fabella may also be involved in or affected by several pathological conditions, including osteoarthritis of the knee involving the fabella, isolated fabellofemoral osteoarthritis, fabella syndrome, common fibular nerve impingement, fabella dislocation, and fractures [5]. Fractures of the fabella are very rare and have only been sporadically reported in the literature [6–13]. We report two cases of fabella fractures in patients who have (very) recently undergone total knee replacement with correction of valgus malalignment.

## 2. Case Presentation

**Figure 2:**
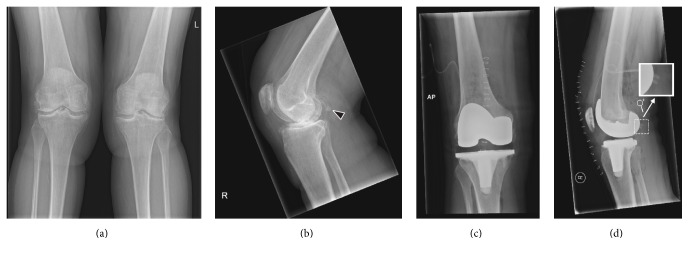
Preoperative (a, b) and immediate postoperative (c, d) knee radiographs in a 68-year-old woman who underwent total knee arthroplasty of the right knee because of symptomatic knee osteoarthritis with valgus malalignment. Preoperative knee radiographs (anterior-posterior view of both knees (a) and lateral view of the right knee (b)) show osteophytes, sclerotic changes, and some degree of generalized joint space narrowing on both sides, in keeping with femorotibial osteoarthritis. There is approximately 10° valgus malalignment of the right knee and approximately 14° valgus malalignment of the left knee. Note the normal appearing fabella ((b) arrowhead). Immediate postoperative radiographs of the right knee on the same day of surgery (anterior-posterior view (c) and lateral view (d)) show not only a good position of the total knee arthroplasty with a well-aligned knee joint but also a horizontal fracture through the fabella with only minimal dislocation ((d) including magnified view of the fabella).

*Case 1. *The first patient who was diagnosed with a fabella fracture was a 68-year-old woman with a history of deep venous thrombosis and pulmonary embolism due to factor V Leiden deficiency and open lateral meniscectomy of the right knee. She presented with recurrent right-sided knee pain and hydrops, and her knee radiographs demonstrated signs of femorotibial osteoarthritis on both sides with approximately 10° valgus malalignment of the right knee and approximately 14° valgus malalignment of the left knee. She underwent total knee arthroplasty of the right knee, using a standard ventral midline incision, medial arthrotomy, and placement of both cemented femoral and tibial and patellar components. Radiography of the right knee immediately after surgery, on the same day, showed not only a good position of the total knee arthroplasty and corrected valgus malalignment but also a fracture of the fabella (Figure 2). Her postoperative course was otherwise uncomplicated, and she was dismissed from hospital seven days after surgery. Further follow-up was also unremarkable.

**Figure 3:**
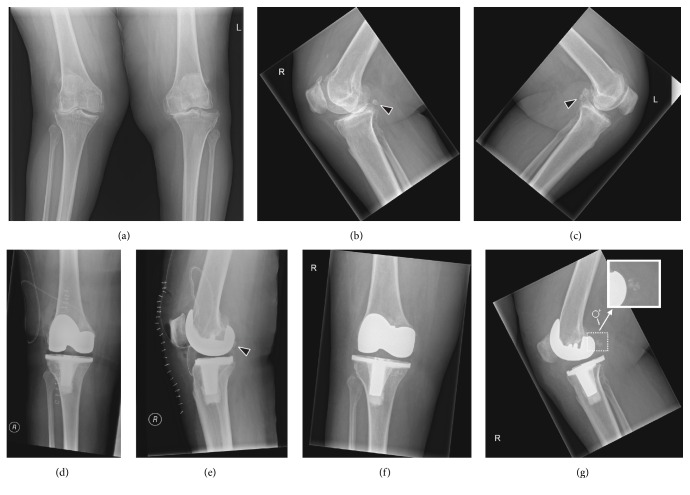
Preoperative (a–c), immediate postoperative (d, e), and 45-day postoperative (f, g) knee radiographs in a 63-year-old woman who underwent total knee arthroplasty of the right knee because of symptomatic knee osteoarthritis with valgus malalignment. Preoperative knee radiographs (anterior-posterior (a) and lateral views (b, c) of both knees) show osteophytes, sclerotic changes, and severe joint space narrowing of right-sided lateral and left-sided medial femorotibial compartments, in keeping with femorotibial osteoarthritis. There is approximately 23° valgus malalignment of the right knee and approximately 7° varus malalignment of the left knee. Note the normal appearing fabellae ((b) and (c) arrowheads). Immediate postoperative radiographs of the right knee on the same day of surgery (anterior-posterior view (d) and lateral view (e)) show a good position of the total knee arthroplasty with a well-aligned knee joint and with a still normal appearing fabella ((e) arrowhead). However, 45-day postoperative radiographs of the right knee (anterior-posterior view (f) and lateral view (g)) show a shattered patella ((g) including magnified view of the fabella).

*Case 2*. The second patient who was diagnosed with a fabella fracture was a 63-year-old woman with a history of obesity, dyslipidemia, hypertension, cardiac failure, gout, and Ménière disease, who suffered from progressive right-sided knee pain. Radiography demonstrated signs of femorotibial osteoarthritis on both sides, with approximately 23° valgus malalignment of the right knee and approximately 7° varus malalignment of the left knee. She underwent total knee arthroplasty of the right knee, using a standard ventral midline incision, medial arthrotomy, and placement of both cemented femoral and tibial components, but without a patellar component. Radiography of the right knee immediately after surgery, on the same day, showed a good position of the total knee arthroplasty and corrected valgus malalignment, with a normal appearing fabella. Three days later she was dismissed from hospital. A repeated radiograph of the right knee 45 days after surgery, however, showed a fractured fabella (Figure 3). Her medical records did not mention any specific circumstances or symptoms that could be related to this fracture. No additional treatment was given because of this fabella fracture. Further follow-up was unremarkable.

## 3. Discussion

The two patients with fabella fractures who have been presented in this report had very recently undergone total knee arthroplasty and had considerable preoperative valgus malalignment of the knee that was corrected intraoperatively. Anatomical and biomechanical factors could play a role in the development of these fractures. The fabella is situated at the endpoint of the oblique popliteal ligament and the lateral gastrocnemius tendon and is connected with the styloid process of the fibular head through the fabellofibular ligament (Figure 1) [15], with all of these structures exposing the fabella to tensile forces. Both patients had preoperative valgus malalignment of the knee, and it can be speculated that its sudden intraoperative correction stresses the abovementioned tendoligamentous structures beyond their adapted normal physiological ranges. Since the fabella is located at the intersection point of these tensile forces, fracture of this sesamoid bone may occur. Particularly the fabellofibular ligament, which is a static stabilizer that tenses when the knee is fully extended [12], is thought to expose the fabella to considerable stress after correction of knee valgus alignment, due to the nearly parallel orientation of its long axis to the main direction of knee motion (flexion extension). The horizontal orientation of the fracture plane through the fabella in the first case (Figure 2) also indicates tensile forces in the craniocaudal direction, which supports this theory. Of interest, the fabellofibular ligament has been reported to be apparently always present when a bony fabella is found [14]. In a human cadaver study, it was shown that the presence of a bony fabella seems to correlate to thickening of the fabellofibular ligament [4]. However, whether this was also the case in the two presented patients remains unclear due to the lack of direct intraoperative or arthroscopic inspection. On another note, the interaction between the fabella and the gastrocnemius muscle is still unclear. Nevertheless, it can be speculated that the fabella plays a role in supporting the mechanical moment of the lateral head of the gastrocnemius muscle, thus increasing tensile forces that may also have led to a fracture of the fabella in the two presented patients. Other than suddenly increased strain of the posterolateral ligamentous complex with subsequent fracture of the fabella, the observed fabellar pathology may, hypothetically, also have been due to osteonecrosis or osteochondritis dissecans. Unfortunately, it was not possible to correlate the radiological observations to pathological findings.

Since the first report in 1932 [6], only a limited number of fabella fractures have been described in the literature, with most cases due to direct trauma to the (postero)lateral knee [6–8, 10, 12, 13], one case that is probably due to chronic microtrauma [9], and three cases associated with total knee arthroplasty [11]. In the report by Theodorou et al. [11], three total knee arthroplasty-related cases of fabella fractures were collected from two institutions over a nine-year period. During the follow-up period after total knee arthroplasty that varied between four months and nine years, all three patients presented with the major complaint of new and increasing pain in the reconstructed knee, with two of the patients also having an altered gait and swelling of the knee [11]. None of the three patients gave a history of trauma or infection [11]. Fabella fractures were radiographically detected in all cases. Theodorou et al. [11] hypothesized that with varus limb alignment or component varus malalignment, as in two of their patients, the concentration of eccentric mechanical loads across the medial aspect of the knee may be associated not only with higher mechanical failure rates but also with strain of the posterior capsuloligamentous complex of the knee that may have caused impingement of the fabella against the components of the prosthesis [11]. Likewise, hyperextension of the knee in the standing stance, as in one of their patients, may have caused impingement of the fabella on the prosthesis components [11]. Increased and chronic accumulation of stress to the fabella may, in turn, lead to a fracture of this sesamoid bone [11]. In addition, excessive knee motion postoperatively, especially in physically active patients, may result in vigorous gastrocnemius contracture that, in a previously compromised fabella, may lead to the development of a fracture [11]. An important difference between Theodorou et al.'s report [11] and the present study is that the fabella fractures in the latter study occurred (almost) immediately after total knee arthroplasty, in the first case on the same day of surgery and in the second case somewhere between one and 45 days after surgery. This may be due to the suddenly increased stress on the posterolateral ligamentous complex, combined with contraction of the gastrocnemius muscle, rather than a more slowly increasing and chronic stress around and on the fabella as in Theodorou et al.'s cases [11]. Thus, orthopedic surgeons and radiologists should be aware of the possibility of fabella fractures immediately after total knee arthroplasty with valgus malalignment correction. However, the clinical consequences of these fractures in this patient population are not completely clear yet. Although fabella fractures may be a cause of severe posterolateral knee pain with physical impairment, the medical records of the two presented patients did not mention any specific symptoms that could be related to these fractures. Such symptoms may have remained unnoticed in the immediate postoperative situation, in which it is usual to experience some degree of pain and physical impairment, and in which analgesics are also administered. It should also be mentioned that experimental and biomechanical studies have suggested the fabellofibular ligament (and arcuate ligament) to be less important for joint stabilization than the popliteal tendon and its associated popliteofibular ligament [4].

In conclusion, fabella fractures are considered very rare, but they can be encountered in patients who have (very) recently undergone total knee replacement with correction of valgus malalignment. Special attention should be paid to the fabella for not missing a fabella fracture in these patients.
